# Prevalence and Factors Associated With Occupational Noise-Induced Hearing Loss Among Palm Oil Mill Workers in Selangor, Malaysia

**DOI:** 10.7759/cureus.66077

**Published:** 2024-08-03

**Authors:** Gowri Mutthumanickam, Rama Krishna Supramanian, Yin Cheng Lim

**Affiliations:** 1 Social and Preventive Medicine, Universiti Malaya, Kuala Lumpur, MYS

**Keywords:** hearing disorder, prevalence, palm oil mill, noise-induced hearing loss, hearing impairment

## Abstract

Introduction

Occupational noise-induced hearing loss (NIHL) continues to be a significant public health issue globally, with Malaysia being no exception. In Malaysia, the majority of NIHL cases are reported from the manufacturing sector, with Selangor among the states with the highest number of confirmed cases. This study aimed to assess the prevalence of and factors associated with occupational NIHL among palm oil mill workers in Selangor, Malaysia.

Methods

A cross-sectional study was conducted to analyze the data from the data collection form, noise risk assessment reports, and audiometric test results done between 2021 and 2022 with a comparable baseline audiometric test.

Results

A total of 143 participants from three palm oil mills joined this study. The prevalence of NIHL was 42.7% (n = 61). Following the logistic regression model, NIHL was significantly associated with a duration of employment of 10 years and above, a history of occupational noise exposure at the previous workplace, and the use of personal hearing protectors at the current workplace with an adjusted OR of 2.41 (95% CI (1.14, 5.07)), 5.89 (95% CI (2.38, 14.53)), and 0.36 (95% CI (0.16, 0.83)), respectively.

Conclusion

The prevalence of NIHL among the study participants was high, and the associated factors are modifiable factors that can be prevented with a comprehensive hearing conservation program in the palm oil mills.

## Introduction

Occupational noise-induced hearing loss (NIHL) remains a major public health concern in all regions of the world, especially in Malaysia [1]. Globally, it has been estimated that 16% of adult-onset hearing loss is due to occupational noise, and more than four million disability-adjusted life years are lost due to NIHL [2]. In Malaysia, most of the NIHL cases reported were from the manufacturing industry, accounting for 87.7% for the year 2021 [3]. Based on the statistics from the Department of Occupational Safety and Health (DOSH), Penang, Johor, Selangor, and Negeri Sembilan were the states with the highest number of confirmed cases from 2018 to 2020. The number of confirmed cases of occupational noise-related hearing disorders in Selangor in 2021 was 221 cases.

Malaysia spent RM7 million to compensate for NIHL cases from 2010 to 2012, with many claims from the manufacturing industries [4]. Apart from affecting the country’s economy, it also has a financial impact on the affected individual and the organization by increasing employee turnover and absenteeism, decreasing productivity, a possible contribution to industrial accidents, and a loss of income due to the loss of workdays [5,6]. Furthermore, workers who are exposed to high levels of noise have detrimental effects on their health and psychosocial well-being. Besides causing auditory effects, it may also cause nonauditory effects such as hypertension, fatigue, headache, tinnitus, anxiety, and depression [7]. The workers’ quality of life is also reduced by affecting their social, emotional, communication, and cognitive functions [8].

In Malaysia, palm oil industries play an important part in the Malaysian economy. Malaysia is among the world’s leading producers and exporters of palm oil after Indonesia, thus making palm oil mills among the noisiest industrial workplaces [7]. To produce crude palm oil, fresh fruit bunches are harvested and transported to mills, which are often located near plantation areas. The fruits are first sterilized using steam before going through the threshing process to separate the fruits from the bunches. To extract crude palm oil, the fruitlets are next moved through the digester and the press machine. Further processing of the squeezed, digested fruit results in the production of fibers, palm shells, and kernels [9,10]. The overall occupational noise exposure in a palm oil mill is above 90 dB(A) [7], which is beyond the noise exposure limit (NEL) of 85 dB(A) set by the Occupational Safety and Health Act 1994.

As more mechanizations are introduced into palm oil mill operations, the risk of workers being exposed to excessive noise has increased over the years. There were a number of studies conducted among palm oil mill workers in Malaysia [9,11]. However, there are no known published studies conducted among palm oil mill workers in Selangor. Therefore, this applied research aims to determine the prevalence and factors associated with NIHL among palm oil mill workers in a major palm oil industry in Selangor.

## Materials and methods

Study design

This was a cross-sectional study conducted from March to August 2023. The sample size calculation was established based on an OR of 5.15 [12]. The sample size required was 199 workers, with an estimation of 20% of the non-response rate. An OR was used for sample size calculation as this is also an association study, and a larger sample size was yielded compared to the calculation using the prevalence.

Participants

**Figure 1:**
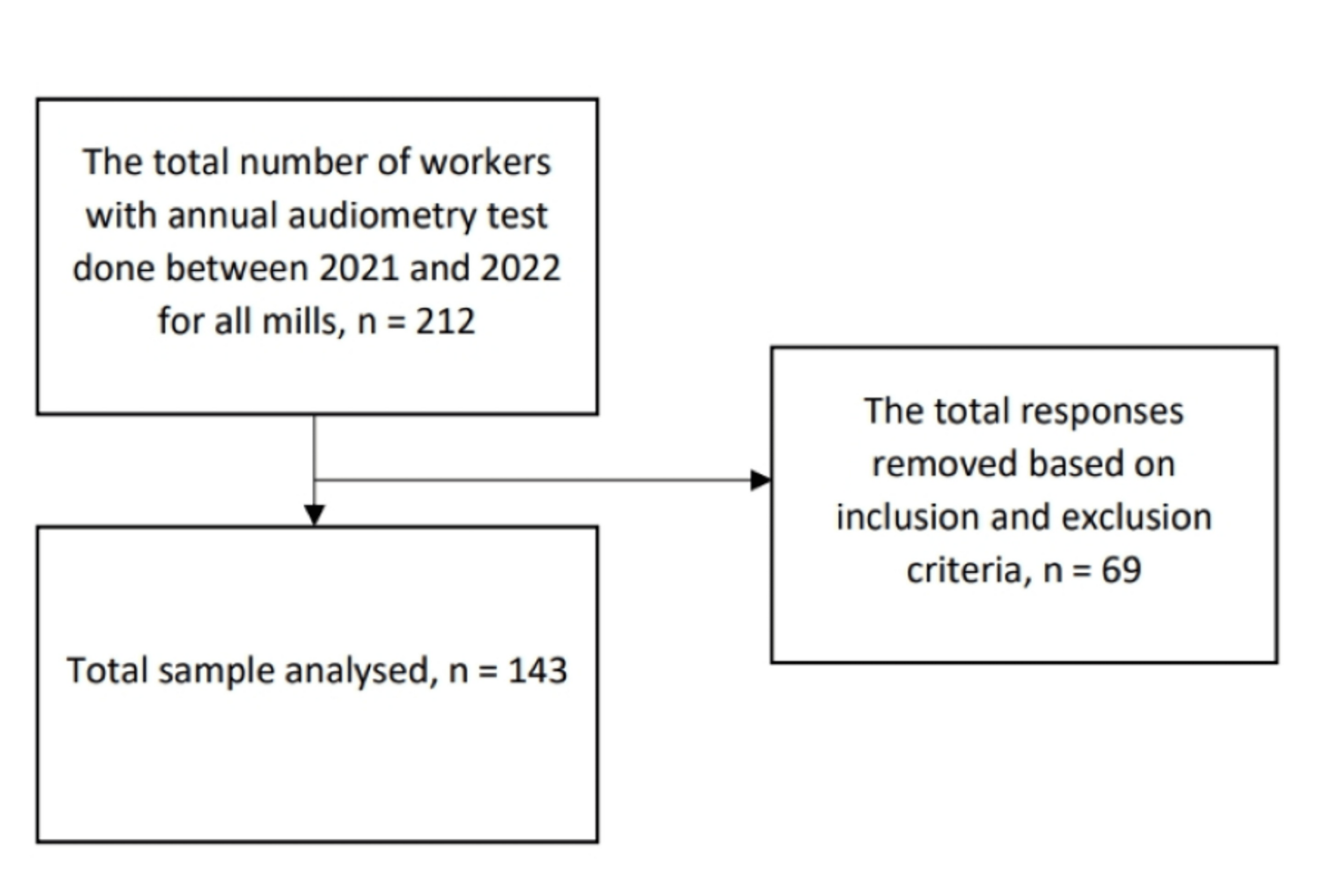
Summary of responses analyzed

Based on the inclusion and exclusion criteria, 143 workers from three palm oil mills in Selangor were included in this study (Figure 1). The inclusion criteria were workers with a duration of employment of at least six months with two audiometry test results, one between 2021 and 2022 and a previous baseline audiometry test result conducted during work commencement, workers and mill managers who are willing to participate and give consent, and participants able to understand either Malay or English. Workers who have been diagnosed by a medical practitioner to have preexisting hearing disorders due to other factors such as ear infections, sustained injuries that would have directly affected their hearing conditions, or had undergone treatment with any ototoxic drugs were excluded from the study.

Data collection

Participants were recruited with the help of an in-charge officer in each palm oil mill. The officer was then briefed on the data collection form, and the forms were distributed to all workers. The data collection form was constructed based on Appendices 4 and 6 of the Industry Code of Practice (ICOP) for Management of Occupational Noise Exposure and Hearing Conservation 2019, which is a legal document used in Malaysia. The data collection form was translated and back-translated from English to Malay to maintain uniformity. The researcher also visited each palm oil mill to collect data from the audiometry test reports and noise risk assessment (NRA) reports and conduct walk-through surveys.

Variables

The dependent variable for this study was NIHL, defined as the audiogram showing patterns of sensorineural hearing loss with “notching” at 3,000, 4,000, or 6,000 Hz and recovery at 8,000 Hz. The hearing threshold must exceed 25 decibels. Pure-tone audiometric rest (PTA) was conducted annually to monitor for hearing disorders among workers exposed to excessive noise above the NEL or workers at risk of developing occupational noise-related hearing disorders. The hearing thresholds were tested for both ears at frequencies of 500 Hz, 1,000 Hz, 2,000 Hz, 3,000 Hz, 4,000 Hz, 6,000 Hz, and 8,000 Hz. The PTA was conducted by trained personnel at registered audiometry test centers. Those workers with abnormal audiograms were then subjected to a medical examination by an occupational health doctor (OHD) and proceeded with a diagnostic audiometry test. The information on the final diagnosis of NIHL was gathered from the Report for Occupational Diseases/Poisoning - Information Required for Noise-related Hearing Disorders (Appendix 6) [5] and the Hearing Assessment Report.

The independent variables were categorized under three domains, which are sociodemographic factors (nationality, gender, and age), lifestyle behavior factors (exposure to recreational noise or hobbies that affect hearing, such as listening to loud music), and occupational noise exposure factors (job and work station categories, duration of employment, intensity of noise exposure at workstations, use of personal hearing protectors (PHPs) at current and previous workplaces, previous history of occupational noise exposure, presence of a hearing conservation program, HCP). This information was obtained through self-reporting by the respondents using the data collection form. The NRA report was reviewed to obtain information on the personal and environmental noise level assessments done at the workplace. Walk-through surveys were conducted to obtain information on the types of jobs, workstations, work processes, and work safety practices.

The HCP was assessed based on five components, which were the NRA, noise reduction measures, audiometry testing, information, instruction, training, and recordkeeping [5]. Based on the walk-through survey and discussion with the employees, none of the mills has fulfilled all the components of a comprehensive HCP. For any missing responses, information was obtained from the respective workers, mill supervisors, preexisting company records, and the NRA reports of the mills.

Data analysis

The data was analyzed using IBM SPSS Statistics for Windows, Version 27.0 (Released 2020; IBM Corp., Armonk, NY, USA). The p-value used was 0.05, and statistically significant data was determined by a p-value of less than 0.05. A binomial logistic regression was used to determine the association between the independent and dependent variables. All the factors were assessed individually using the simple logistic regression model. Subsequently, factors with a p-value of less than 0.25 or clinically significant were included in the multiple logistic regression to adjust for confounders. This is to ensure that significant independent variables were not accidentally removed while conducting the multivariable analysis [13]. The associations were presented by the OR and 95% CI. The forward likelihood ratio method was used in the binary logistic regression. The final model was considered for independent variables with a p-value of less than 0.05. Multicollinearity was checked for the independent variables, and the final model was assessed for goodness of fit using the Hosmer-Lemeshow test and area under the receiver operating characteristic (ROC) curve. The recommended area under the ROC curve is more than 0.70 for the goodness of fit assumption to be met [13].

## Results

The total sample size achieved was 143, with a response rate of 100%. The prevalence of NIHL among palm oil mill workers was 42.7% (n = 61).

Table 1 details the characteristics of the participants. Most workers were Malaysian (83.2%, n = 119) and male (84.6%, n = 121). Approximately 51% (n = 73) were under 40 years old, with a mean age of 40.87. The majority held non-supervisory positions (81.1%, n = 116) and worked in the mill or processing units (77.6%, n = 111). The mean duration of employment was 12.52 years, with 51.7% (n = 74) having worked for less than 10 years. The average noise exposure at workstations was 84.9 dB(A), with 53.8% (n = 77) working in areas where noise exposure was less than 85 dB(A). Most workers reported using personal hearing protection (66.4%, n = 95). Among the workers who had previously been in noisy environments (24.5%, n = 35), 14.7% (n = 21) had used personal hearing protection. In contrast, 75.5% (n = 108) had not worked in noisy environments, making the use of personal hearing protection not applicable, and this variable was not analyzed further. Most workers reported that no HCP was present at their workplace (89.5%, n = 128) and that 65% (n = 93) were not exposed to any recreational noise.

**Table 1:** Sociodemographic, occupational noise exposure, and lifestyle behavior factors of study participants (n = 143) HCP, hearing conservation program; PHP, personal hearing protector

Variables	Frequency (%)	Mean (SD)
Sociodemographic factors		
Nationality		
Malaysian	119 (83.2)	
Non-Malaysian	24 (16.8)	
Gender		
Male	121 (84.6)	
Female	22 (15.4)	
Age		40.87 (10.985)
<40 years old	73 (51.0)	
≥40 years old	70 (49.0)	
Occupational noise exposure factors		
Job categories		
Non-supervisory	116 (81.1)	
Supervisory	27 (18.9)	
Workstation categories		
Mill/processing unit	111 (77.6)	
Office	32 (22.4)	
Duration of employment		12.52 (10.608)
<10 years	74 (51.7)	
≥10 years	69 (48.3)	
Noise exposure (dB(A))		84.9 (6.572)
<85 dB(A)	77 (53.8)	
≥85 dB(A)	66 (46.2)	
Current use of PHPs		
Yes	95 (66.4)	
No	48 (33.6)	
Previous history of occupational noise exposure		
No	108 (75.5)	
Yes	35 (24.5)	
Previous use of PHPs		
Yes	21 (14.7)	
No	14 (9.8)	
Not applicable	108 (75.5)	
HCP carried out		
No	128 (89.5)	
Yes	15 (10.5)	
Lifestyle behaviors factors		
Lifestyle behaviors		
No	93 (65.0)	
Yes	50 (35.0)	

Table 2 explains the association between sociodemographic, occupational noise exposure, and lifestyle behavior factors with NIHL. In the univariate analysis, seven variables were found to have a p-value of less than 0.25 and were included in the multivariable analysis.

**Table 2:** Univariate and multivariate analysis of NIHL among noise-exposed workers in palm oil mills AOR, adjusted OR; HCP, hearing conservation program; NIHL, noise-induced hearing loss; PHP, personal hearing protector

Variables	Univariate analysis: unadjusted OR (95% CI)	Univariate analysis: p-value	Multivariate analysis: aOR (95% CI)	Multivariate analysis: p-value	
					Sociodemographic factors					
					Nationality					
Malaysian	Reference				
Non-Malaysian	0.47 (0.19, 1.28)	0.148			
Gender					
Female	Reference				
Male	1.09 (0.43, 2.74)	0.857			
Age					
<40 years old	Reference				
≥40 years old	2.92 (1.47, 5.80)	0.002			
Occupational noise exposure factors					
Job categories					
Supervisory	Reference				
Non-supervisory	1.33 (0.56, 3.16)	0.513			
Workstation categories					
Office	Reference				
Mill/processing unit	1.57 (0.69, 3.55)	0.284			
Duration of employment					
<10 years	Reference				
≥10 years	2.72 (1.37, 5.39)	0.004	2.41 (1.14, 5.07)	0.021	
Noise exposure (dB(A))					
<85 dB(A)	Reference				
≥85 dB(A)	1.75 (0.90, 3.42)	0.101			
Current use of PHPs					
Yes	Reference				
No	0.36 (0.17, 0.77)	0.008	0.36 (0.16, 0.83)	0.016	
Previous history of occupational noise exposure					
No	Reference				
Yes	6.03 (2.55, 14.22)	<0.001	5.89 (2.38, 14.53)	<0.001	
HCP carried out					
Yes	Reference				
No	3.31 (0.89, 12.31)	0.073			
Lifestyle behaviors factors					
Lifestyle behaviors					
No	Reference				
Yes	1.23 (0.62, 2.47)	0.554			

After running the multivariable analysis, three factors from the occupational noise exposure characteristics were significantly associated with NIHL after controlling for confounders. The first factor, workers working for 10 years and above, had 2.41 odds with a 95% CI (1.14, 5.07) of developing NIHL. Next, workers with a previous history of occupational noise exposure were 5.89 times more likely to develop NIHL, with a 95% CI (2.38, 14.53). The third significant factor was using PHP in the current workplace. Workers using PHP at their current workplace were 0.36 less likely to develop NIHL, with a 95% CI (0.16, 0.83).

Next, multicollinearity between the independent variables in the final model was checked. The tolerance value was more than 0.1, and the variance inflation factor was less than 5; thus, the independent variables were not correlated. The final model was assessed for goodness of fit using the Hosmer-Lemeshow test (p-value 0.256) and area under the ROC curve (0.74 with 95% CI (0.66, 0.82)), suggesting that this model fits the data.

## Discussion

The prevalence of NIHL in this study was 42.7% (n = 61) and was slightly lower compared to a study done among steel industry workers in Egypt, which was 47% (n = 186). This study used a similar operational definition as ours, where NIHL was defined as a notch shown at 4 kHz (around 3-6 kHz) [14]. There was another study conducted among palm oil mill workers in the Eastern region of Peninsular Malaysia, which has a prevalence of NIHL of 50.8% (n = 251). However, they defined NIHL as bilateral high-frequency hearing loss (3,000-6,000 Hz) with or without an audiometric notch [9]. This study showed that there was a crude association between the age category of 40 years old and above with NIHL. The unadjusted OR value was 2.92 with a 95% CI (1.47, 5.80). However, upon conducting a multivariable analysis, it was no longer found to be significant, possibly due to its confounding effect. Studies have shown that the risk of hearing loss increases with age, especially at higher frequencies, and age factors always behave as confounders in risk analyses [12,15,16].

Based on the multivariable analysis, there were three factors found to be significantly associated with NIHL. The duration of employment of 10 years and above was found to be 2.41 times more likely to cause NIHL, with a 95% CI (1.14,5.07). This adjusted OR (aOR) was similar to two other previous studies, which were 2.92 (95% CI (1.16, 7.33)) [12] and 2.50 (95% CI (1.4,4.7)) [17]. Besides that, a few other systematic reviews also found significant associations between longer durations of employment with NIHL [18-21]. The second factor was a history of occupational noise exposure at the previous workplace, with an aOR of 5.89 (95% CI (2.38,14.53)). There was no known published study that found an association between a history of occupational noise exposure at the previous workplace to NIHL. However, there were multiple systematic reviews [18,19,21] and studies [9,12,14,22-24] that found an association between occupational noise exposure and hearing loss. Finally, workers using PHPs at their current workplace were found to be 64% less likely to develop NIHL. The aOR was 0.36 (95% CI (0.16,0.83)). The finding from our study was similar to another study conducted among metal workshop workers in Ethiopia (aOR = 0.3; 95% CI: 0.1, 0.6) [23]. Similarly, infrequent use or not using PHP was found to be a significant risk factor in developing NIHL [20,21].

Even though in this study the intensity of noise exposure was studied and found to be significant in the univariate analysis, there was no significant association found in the multivariable analysis. This might be due to the small sample size, which was supported by the post hoc power analysis of 37.31%. Therefore, the nonsignificant association in the multivariable analysis might be attributed to this insufficient power. The presence of an HCP was also found to not be significantly associated with NIHL. Theoretically, HCP is known to reduce the occurrence of NIHL [5]. This was supported by another finding in this study, which was that those wearing PHP were less likely to develop NIHL. The use of PHP in the workplace is also a component of the HCP. Nevertheless, upon conducting post hoc power analysis, the study was underpowered at 76.74%, thus there was no significant association between HCP and NIHL.

Based on the walk-through survey conducted, there was no proper HCP in the mills. There was one mill that had a trained occupational safety and health (OSH) coordinator who oversaw the safety practices in the mill. In this mill too, there was a specific administrative control where others could report on workers not complying with the OSH standards through an online system, and actions towards these workers were taken. This effort increases compliance with the OSH standards in the mill. Upon interviewing the supervisors and employees, it was noted that all the workers were provided with PHP regardless of their workstations, as they would need to be wearing PHP upon entering the “hearing protection zone." However, some workers were not compliant as it interfered with communication and hearing warning signals that can lead to industrial accidents. Based on the hierarchy of control, the use of PHP is the least effective measure, as any misuse leads to workers being exposed to harmful levels of noise. Furthermore, the workstations were near each other, thus affecting other workers too.

Therefore, more objective measures such as engineering control to reduce noise at the source and administrative control are more effective measures for reducing the noise level at workstations. In addition, DOSH has also published the ICOP for Management of Occupational Noise Exposure and Hearing Conservation 2019 to help industries comply with the regulation by guiding excessive noise identification, risk assessment, and the implementation of appropriate control measures at the workplace.

The data used in this study were actual industrial data from the NRA reports and workers’ audiometry test reports. Thus, this applied research guides employers to find practical solutions to address the disease burden. Next, this research studied multiple associated factors for NIHL, ranging from sociodemographic, occupational noise exposure, and lifestyle behavior factors. The study design was a cross-sectional study carried out within a limited time frame to provide timely results to the related stakeholders and utilize the findings for early interventions to prevent NIHL occurrence and progression. Engaging with the respective stakeholders throughout the study also ensured the findings were relevant and increased the chance of being utilized by them to benefit the organization as well as the workers.

Limitations

The study faced several limitations. As a cross-sectional study, it could not establish causal relationships between the associated factors and NIHL. Due to limited resources, the study was restricted to three palm oil mills in Selangor, resulting in a small sample size and the use of universal sampling rather than other probability sampling methods. Despite this, the large effect sizes identified three significant variables associated with NIHL, which can guide the development of comprehensive HCP in mills.

Selection bias was a concern, as workers who did not attend their annual audiometry tests were excluded from the study. To mitigate this bias, the study included the most recent annual audiometry reports with the highest number of participants. Measurement bias was addressed by regularly calibrating the devices used for hearing threshold measurement, personal and area noise monitoring, and interpreting results. Audiometric tests were conducted by trained personnel; results were interpreted by an OHD; and NRA was carried out by a registered noise risk assessor under the DOSH.

Finally, the findings of this study may not be generalizable to all palm oil mills and are specific to the study population.

## Conclusions

This study revealed a high prevalence of NIHL among the participants. Significant factors associated with NIHL included a duration of employment of 10 years or more, a history of occupational noise exposure at previous workplaces, and the use of PHP at the current workplace. These factors are modifiable, suggesting that NIHL can be prevented through the implementation of a comprehensive HCP in palm oil mills. Employers should ensure the program’s success through regular supervision and evaluation by trained OSH coordinators. Additionally, employers must enforce regulations and ensure compliance with OSH standards.

For future research, a larger sample size that includes more palm oil mills should be employed to better understand the prevalence and factors associated with various occupational noise-related hearing disorders, including hearing impairment and permanent standard threshold shift. Interventional studies are also needed to assess the impact of comprehensive HCPs on reducing the prevalence of NIHL among palm oil mill workers.
